# “I just keep quiet about it and act as if everything is alright” – The cascade from trauma to disengagement among adolescents living with HIV in western Kenya

**DOI:** 10.1002/jia2.25695

**Published:** 2021-04-10

**Authors:** Leslie A Enane, Edith Apondi, Mark Omollo, Judith J Toromo, Salim Bakari, Josephine Aluoch, Clemette Morris, Rami Kantor, Paula Braitstein, J Dennis Fortenberry, Winstone M Nyandiko, Kara Wools‐Kaloustian, Batya Elul, Rachel C Vreeman

**Affiliations:** ^1^ The Ryan White Center for Pediatric Infectious Disease and Global Health Department of Pediatrics Indiana University School of Medicine Indianapolis IN USA; ^2^ Academic Model Providing Access to Healthcare (AMPATH) Eldoret Kenya; ^3^ Moi Teaching and Referral Hospital Eldoret Kenya; ^4^ Indiana University‐Purdue University‐Indianapolis Indiana University Indianapolis IN USA; ^5^ Division of Infectious Diseases Department of Medicine Brown University Apert Medical School Providence RI USA; ^6^ Department of Epidemiology Indiana University Fairbanks School of Public Health Indianapolis IN USA; ^7^ Dalla Lana School of Public Health Division of Epidemiology University of Toronto Toronto ON Canada; ^8^ Department of Medicine College of Health Sciences School of Medicine Moi University Eldoret Kenya; ^9^ Division of Adolescent Medicine Department of Pediatrics Indiana University School of Medicine Indianapolis IN USA; ^10^ Department of Child Health and Pediatrics College of Health Sciences School of Medicine Moi University Eldoret Kenya; ^11^ Division of Infectious Diseases Department of Medicine Indiana University School of Medicine Indianapolis IN USA; ^12^ Department of Epidemiology Mailman School of Public Health Columbia University New York NY USA; ^13^ Department of Health System Design and Global Health Icahn School of Medicine at Mount Sinai New York NY USA; ^14^ Arnhold Institute for Global Health New York NY USA

**Keywords:** child, adolescent, retention in care, patient dropouts, psychological trauma, mental health

## Abstract

**Introduction:**

There are approximately 1.7 million adolescents living with HIV (ALHIV, ages 10 to 19) globally, including 110,000 in Kenya. While ALHIV experience poor retention in care, limited data exist on factors underlying disengagement. We investigated the burden of trauma among disengaged ALHIV in western Kenya, and its potential role in HIV care disengagement.

**Methods:**

We performed in‐depth qualitative interviews with ALHIV who had disengaged from care at two sites, their caregivers and healthcare workers (HCW) at 10 sites, from 2018 to 2020. Disengagement was defined as not attending clinic ≥60 days past a missed scheduled visit. ALHIV and their caregivers were traced through phone calls and home visits. Interviews ascertained barriers and facilitators to adolescent retention in HIV care. Dedicated questions elicited narratives surrounding traumatic experiences, and the ways in which these did or did not impact retention in care. Through thematic analysis, a conceptual model emerged for a cascade from adolescent experience of trauma to disengagement from HIV care.

**Results:**

Interviews were conducted with 42 disengaged ALHIV, 34 caregivers and 28 HCW. ALHIV experienced a high burden of trauma from a range of stressors, including experiences at HIV disclosure or diagnosis, the loss of parents, enacted stigma and physical or sexual violence. A confluence of factors – trauma, stigma and isolation, and lack of social support – led to hopelessness and depression. These factors compounded each other, and resulted in complex mental health burdens, poor antiretroviral adherence and care disengagement. HCW approaches aligned with the factors in this model, suggesting that these areas represent targets for intervention and provision of trauma‐informed care.

**Conclusions:**

Trauma is a major factor underlying disengagement from HIV care among Kenyan adolescents. We describe a cascade of factors representing areas for intervention to support mental health and retention in HIV care. These include not only the provision of mental healthcare, but also preventing or addressing violence, trauma and stigma, and reinforcing social and familial support surrounding vulnerable adolescents. In this conceptualization, supporting retention in HIV care requires a trauma‐informed approach, both in the individualized care of ALHIV and in the development of strategies and policies to support adolescent health outcomes.

## 1 INTRODUCTION

There are 1.7 million adolescents living with HIV (ALHIV, ages 10 to 19) globally, including approximately 110,000 in Kenya [1]. ALHIV experience higher rates of care disengagement compared to other age groups, placing them at risk for poor outcomes [2, 3, 4, 5].

Risk factors for disengagement from HIV care may be heterogeneous, ranging from housing needs and poverty, to stigma and psychological challenges [4, 6, 7, 8, 9, 10]. Adolescents are particularly affected by disclosure issues, stigma, mental health, family‐level factors and transitions in care [4, 6, 7, 8, 9, 10]. There are limited data regarding factors underlying disengagement for ALHIV [6, 7, 9, 10, 11].

A growing body of literature has examined the intersection of trauma and HIV risk and treatment outcomes in a range of populations [12, 13, 14, 15, 16, 17, 18]. Very few studies have examined trauma histories and care engagement among ALHIV. Among hospitalized ALHIV in Kenya, trauma was prominent in narratives of disengagement [6]. Among ALHIV in Malawi, witnessing or experiencing violence in the home was associated with decreased ART adherence and missed clinic visits [19].

In this study, we sought to examine the burden of trauma among disengaged ALHIV in western Kenya and to investigate its potential role in HIV care disengagement. We defined traumatic events broadly as potentially life‐threatening or highly stressful experiences which undermine a sense of safety [20, 21, 22]. Trauma may result not only from experiences of violence or injury, but also of loss of loved ones, severe illness or discrimination [20, 21, 23, 24]. To investigate the complex processes by which trauma may contribute to disengagement from care, we undertook a qualitative approach, through in‐depth interviews with ALHIV who had disengaged from care, their caregivers and healthcare workers (HCW).

## 2 METHODS

### 2.1 Setting and study population

This qualitative study was performed in western Kenya. The Academic Model Providing Access to Healthcare (AMPATH) is a comprehensive healthcare programme established by Moi University and Indiana University involving a consortium of several partner institutions and the Ministry of Health in Kenya [25, 26, 27, 28]. The Rafiki Center for Excellence in Adolescent Health at Moi Teaching and Referral Hospital (MTRH) in Eldoret provides adolescent‐friendly services to adolescents with and without HIV infection.

Multidisciplinary HCW serving ALHIV in AMPATH include clinical officers, nurses, outreach workers, social workers, psychologists and peer mentors. When ALHIV miss appointments, outreach workers intensively follow them up through phone calls and home visits [29, 30]. Counselling for adherence, disclosure support or psychological distress may be provided by clinical officers, peer mentors and/or by a psychologist.

### 2.2 Recruitment and enrolment

HCW were purposively sampled to include those managing ALHIV in a range of roles and clinical sites: MTRH (4 clinics), Kitale, Turbo, Webuye, Burnt Forest, Mosoriot and Chulaimbo. Sites were chosen for representativeness of a range of geographic and clinical settings and of availability of adolescent‐friendly services. HCW were recruited from October 2018 to January 2019.

ALHIV were recruited if they were ages 10 to 19 at their last clinic visit; previously in care at MTRH or Kitale; attended ≥1 visit in the 18 months prior to data collection and had not attended clinic ≥60 days past their last scheduled visit. ALHIV and their caregivers were traced by community health volunteers and the study team, through phone calls and home visits. Participants were excluded if tracing revealed they had transferred care without any period of disengagement. Adolescents and caregivers were recruited from May 2019 to March 2020.

### 2.3 Ethics

Standard human subjects’ protections were used to maximize participation in the consent process, minimize any perception of coercion and maintain confidentiality. All participants provided informed consent. Adolescent minors provided assent and their primary caregiver gave consent. Participants were cautioned that some interview questions could be sensitive and that they did not have to answer any questions that made them uncomfortable. Adolescents who disclosed mental health needs during the interview were assessed and provided counselling by a trained and experienced peer mentor, and/or referred to a psychologist. The HIV care programme liaised with all ALHIV to re‐engage them.

A separate interview guide was used for adolescents who had not disclosed awareness of their HIV status, which did not mention HIV. Disclosure status was ascertained through the use of a disclosure screening tool [6].

The study protocol was approved by the Institutional Research and Ethics Committee constituted jointly by Moi University College of Health Sciences and MTRH, and by the Institutional Review Board at Indiana University.

### 2.4 Semi‐structured interviews

**Figure 1:**
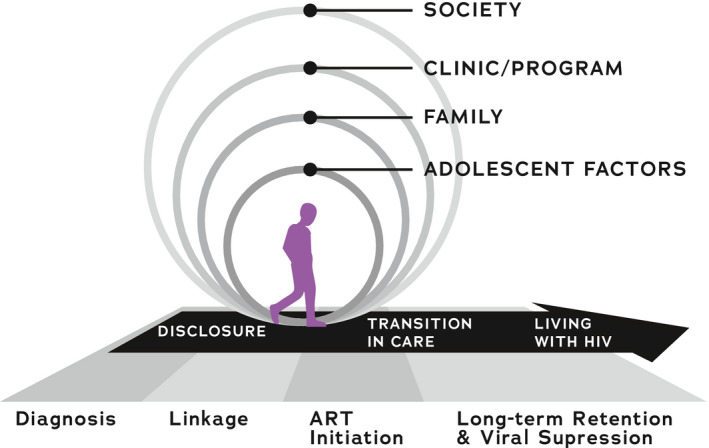
Overarching socio‐ecological framework guiding in‐depth interviews in this study. Permission obtained for re‐printing [6].

Semi‐structured interview guides were developed according to the research questions and a socio‐ecological framework adapted by our team (Figure 1) [6].

Our adaptation recognized the dynamic trajectories of adolescent development, adolescent HIV management and the care cascade, and was informed by our previous work in this area [4, 6, 7, 8]. Questions were open‐ended, and investigated barriers and facilitators to retention in HIV care, and factors underlying disengagement. Dedicated questions assessed for potential role(s) of trauma, stigma or psychological distress. Questions were developed and discussed among multiple team members including native speakers of Swahili to ensure clear interpretation and sensitivity. Traumatic events were queried as “difficult experiences that have caused you stress.” Subsequent probes inquired about examples of potentially traumatic experiences, such as the severe illness or death of a parent, relative or peer; severe illness of the adolescent; learning about one’s HIV status in a traumatic way; or witnessing or experiencing violence, or threats of violence.

Interviews were conducted by research staff fluent in Swahili and English, with training and experience in qualitative interviews, and extensive experience working with ALHIV. The team included a trained peer mentor with experience in mental health counselling, who had not had any prior involvement with study participants. At the conclusion of interviews, the team spent time with participants to discuss any issues that arose and approaches or resources to address them, including through immediate counselling by the peer mentor, and/or referral to a psychologist. All participants were followed up to assess any ongoing needs.

Interviews were audio‐recorded, translated/transcribed into English (single process), by either a professional translator/transcriber or the study team. Every transcript was verified by a second team member reviewing the audio and the transcript.

### 2.5 Analysis

A codebook was developed and organized around the research questions and according to our adapted socio‐ecological framework [6]. Analysis of initial transcripts facilitated elaboration of the codebook, which was iteratively refined through discussion. Several transcripts were then coded by multiple independent coders to establish inter‐rater reliability and consensus. All transcripts were then independently coded by multiple team members using the Dedoose platform. Detailed findings were recorded in memos and discussed. Reports of codes were generated for further interrogation of the data specifically around narratives of trauma and disengagement, which took an inductive approach. Through close reading and re‐reading of the data and triangulation of perspectives (adolescent, caregiver, HCW), overarching themes emerged, and an overall conceptual model was developed for the cascade of factors from trauma to disengagement. The model was discussed among the study team, which included experts in adolescent HIV care and mental health, and shared in group discussions with HCW and with peer mentors (separately). Consensus was reached on the final model. Findings were summarized, including the content of the model, and emergent themes. Representative abbreviated excerpts were selected to illustrate these themes.

## 3 RESULTS

Data were collected from 42 traced, disengaged ALHIV, whose mean age was 17.0 years (range 12.9 to 20.9), and who were predominantly female (62%), orphaned (mother and/or father deceased, 67%) and food‐insecure (60%). Thirty‐four of their caregivers also participated; mothers [16], fathers [2], aunts [4], uncles [3] grandmothers [3], siblings [3] and guardians [3]. In addition, 28 HCW at 10 clinics participated: clinical officers [11], outreach workers [8], nurses [5], social workers [3] and psychologist [1]. HCW were predominantly female (64%), averaged age 37 years (range 23 to 60), and had a median 10 years’ experience in HIV care.

### 3.1 High burden of self‐reported trauma

Disengaged ALHIV reported a heavy burden of trauma from a range of experiences, which continued to impact their engagement in HIV care (Table 1).

**Table 1:** Excerpts of narratives of trauma and its impacts on HIV care retention through a cascade of factors

Speaker	Narrative
High burden of self‐reported trauma	
20 to 24yo F	“This boy… locked me in a room because I told him I was positive. … He called his friends,… he said, ‘didn’t you want to infect us, now infect us.’ You know they raped me, and they were three. So, that’s why I usually really get scared when I date someone and tell him that I am positive.”
Outreach worker, M	“We followed [a sexual abuse] case until the [father] was jailed. The mother stopped clinic, the other girl stopped, and the one comes on and off. She comes when she's very sick, after picking up, she goes away… TB has elapsed twice now. When she comes, she's treated, when she's upright, she goes missing. [It’s] that trauma.… Any time [such cases] come we try to talk, the nurse tries talk, the clinician tries to talk, because we know them and we know the circumstances how they came to care.”
Nurse, F	“We had a young man who [was sexually abused by] a Catholic priest… He used to feel bad especially when he gets here to [clinic]. It's like the whole thing comes back… When it came to the time to start ARVs,… it was like now the reality is hitting him once again. … We really struggled. He would just come, even when you talk to him, he would just be mute. At times he would get emotional. So we knew him. When we see him, we just pick him and skip the queue and finish with him in the shortest time and he goes.”
15 to 19yo F	“We [tested] and he found out he has the disease, and I also have. So, he called my mom and told her he will kill himself or kill me. … If he gets [my ART] he throws them away… I can’t tell him I’m going [to clinic for] myself, I say I am taking the child. [If I tell him I am going to clinic] he always gets offended and angry… Maybe it’s transport he will refuse to give me. I always say I am bringing the child and he facilitates.”
Stigma and isolation	
Caregiver of 15 to 19yo F	“In school, some pupils tell her, ‘Go away, your parent died of that disease and you have it, too’… At times she thinks about it badly. She loses interest for coming to the clinic.”
20 to 24yo F	“That stigma made me feel so bad, especially the fact that it came from my best friend. I even left school. I tried to commit suicide twice, but it did not work.”
Lack of social support	
Clinical officer, M	“[Some caregivers of orphans] – they are not caring enough, they give up making efforts to [support care]… Most of them are like, ‘You take your drug whether you like it or not, it is your life. You will die like the way your parents died.’… Some even have the audacity to tell them when we are with [the] patient here. ‘You don't take your drugs, you will die like the way your mother died of HIV.’ It is so disturbing, it’s so traumatizing…. I had to tell the boy to wait outside, I talk to her. Then we told her, ‘No one chooses to be born with HIV. No one chooses at all.’”
15 to 19yo F	“The other girls said, ‘We don’t want to be walking around with anyone that has HIV.’ I will infect them, I don’t know what… Whenever I went to talk with someone, they would not talk to me because obviously they had been told… I would stay in the house and when I got tired, I would sleep. When I got tired sleeping, I would wake up. My job was to sleep and wake up, like that. … I was being given advice by the doctor. They told me that even if they talk about me, I should pretend that I am not listening to them, let it come in through here and go out the other side.”
Depression & suicidality	
20 to 24yo F	“When I told mom [I needed to come to clinic], she said, no you won’t come. I felt like the world had come to an end, I felt like committing suicide, and I don’t know why that thing comes to my mind when I am stressed. … Since that time, I never came [to clinic].”
20 to 24yo M	“Having stress [prevented me from coming to clinic]. That question you would ask yourself, ‘why me? Why did it happen to me?’ There is even a time that I said to myself, let me care less, if I will die, let me die. I had reached the end. I could not stay peacefully with others. My conscience judged me… [Around others] I was thinking about many things. … Mostly about how they live… You know they had some kind of strength which when I could try to compare with mine, I was like the weakest person. So, I isolated myself … I was just seeing myself as different from other people.”
Clinical officer, M	“They feel the inferiority complex as in with having HIV you cannot be empowered, your fate is sealed, as in it is just death that is waiting for you… Sometimes it becomes serious, as in, ‘Why should I come for care?’ It can be that bad. It can be very bad.”
Addressing trauma in adolescent HIV care	
Clinical officer, M	“The ones who don’t like talking [about trauma] are likely to disengage, because it is a kind of a time bomb… They can be adherent to their treatment well, but you just know that something is not right. So, you just try your best but sometimes it is not enough or you have done enough but really they just disengage from care.”
Nurse, F	“Most of the time the staff did not know how talk to a child, we didn't even think it was an important question to ask if he or she is facing difficulties and things like that just pass by unnoticed at the clinic because all you see is a happy child, taking their medication. [A research study] helped us realize that poor adherence can come as a result of trauma the child faced, maybe violence and stress at their home and things like that, so it really helped us probe the children on what they have been going through.”

Some adolescents described learning their status (through disclosure or testing) as a traumatic experience. This was particularly difficult for orphaned adolescents coping with both the HIV diagnosis and the loss of their mother. In the words of one adolescent, “not being able to know my mother” was a source of ongoing stress. For these adolescents, HIV diagnosis and treatment was associated with this traumatic loss.

*“She kept on asking me and then I told her, ‘you got sick and it is because of your parents who caused it.’ She asked me, ‘what illness am I suffering from?’ I told her, ‘you are sick from HIV.’ She asked me, ‘will I die like my mother?’”* – Caregiver of 15 to 19yo F

A parallel finding was that for adolescents who tested positive for HIV after sexual assault, HIV care was associated with sexual trauma. Histories of sexual assault were frequently disclosed by ALHIV. For one adolescent, trauma from sexual assault was compounded by the loss of a pregnancy.

*“When I found I had the disease [after a sexual assault], they said I start treatment there; but I had not believed, I was alone. … I threw the drugs and stayed away*.*”* – 15 to 19yo F

Whether associated with parental loss, or sexual violence, for these adolescents, taking ART or attending a clinic brought memories of the trauma.

*“Anytime they look at those drugs, some of them would recall the incident so it triggers their worst out of their experience*.*”* – Clinical officer, M

Care was directly impacted by ongoing intimate partner violence, or by family‐level conflicts, including enacted stigma, or emotional or physical abuse.

*“The new dad saw the HIV‐positive child as a burden… he started stigmatizing this child until he got to a point of beating him. This child was wishing if they could get out of their home,… but the mother did not want anybody to know…. So, in hiding, what was happening in the family, it made the child refuse to take medication completely… Not taking medication for a whole year took a toll on him and we lost him. [He passed on.]”* – Nurse, F

Other forms of trauma described by adolescents included severe illness in themselves or others – “when I got TB, I felt the world was ending.” Several adolescents had experienced physical violence, including at home or in the region, as during the post‐election crisis in 2007 to 2008. Street‐connected youth experienced intractable poverty, very little social support, and the loss of their peers.

*“The adolescents themselves, when they lose one of their own they get so down.… They say, ‘Hey, so we shall be next? So, who is next?’”* – Outreach worker, F

### 3.2 Stigma and Isolation

Most adolescents reported experiences of enacted stigma, which were themselves traumatic and directly contributed to disengagement. Stigma was enacted by peers, teachers, relatives, neighbours or others.

*“In school, some pupils tell her, ‘Go away, your parent died of that disease and you have it, too’. … At times she thinks about it badly. She loses interest for coming to the clinic.”* – Caregiver of 15 to 19yo F

Adolescents described internalized stigma and isolation which made them less able to seek support for coping with trauma. Beyond HIV stigma, adolescents experienced stigma surrounding sexual assault or domestic violence. These contributed to isolation and worsened mental health.

*“I feel I am very different from [my friends] because ever since I was young, I had it. … I feel like I am not a human being*. *I mean I am nothing. I don’t know.”* – 15 to 19yo F

### 3.3 Lack of social support

Some adolescents lacked sufficient support in their close relationships to engender resilience despite trauma, stigma and isolation. Furthermore, they lacked support for HIV care, such as caregiver support for adherence, or resources or skills to navigate HIV care and facilitate retention. Adolescents who experienced orphanhood; family conflicts or neglect; poverty or being street‐connected faced significant instability and barriers to care. As a result, it was seemingly untenable for adolescents to cope and to navigate care for HIV or trauma.

*“It is very difficult for you to think about care when you don’t have a shelter, when you don’t have a place to put your head,… you don’t have regular meals, you don’t have somebody to look up to because everyone you see around who has HIV has already died. So, if they don’t have a proper psycho‐social support system, most of them end up being lost to follow‐up.”* – Clinical officer, M

### 3.4 Depression and suicidality

Multiple traumas and experiences of stigma were compounded by isolation from internalized stigma or abuse, resulting in hopelessness, depression and suicidality. Hopelessness was central to decreased adherence and disengagement. One outreach worker described, “it all goes back to giving up on life. Especially if there’s no parent, [the adolescent thinks,] ‘why am I living if they died?’” A clinical officer described following up an adolescent that disengaged from care after witnessing domestic violence – “he said he had lost hope in life.”

*“One problem goes and another one comes; they have never come to an end… When someone does something bad to me, I just accept, just because there is nothing I can do. But I try to tell [peer mentor] mostly … if [peer mentor] can’t handle it, I tell another person. But if I feel it is that much of a secret, I just keep quiet about it and act as if everything is alright*.*”* – 20 to 24yo F

### 3.5 Addressing trauma in adolescent HIV care

HCW described intense management of adolescents dealing with a range of trauma, particularly sexual and physical violence. These cases were socially complex and involved efforts of the full care team. Trauma presented a major barrier to retention, particularly for adolescents with ongoing mental health needs. HCW emphasized being attuned to the possibility of trauma, including among adolescents who outwardly project stability or who adhere well to ART.

HCW described several approaches to adolescent trauma. They involved law enforcement (in criminal situations); re‐located adolescents and intervened in family conflict. They provided counselling and linkages to peer support, and psychologist referral. When needed, these efforts were coupled with financial assistance, social work support or family counselling. HCW met adolescents coping with trauma on their terms, made flexible appointment times, and saw them quickly if being at the clinic was itself traumatic.

HCW observed that while some ALHIV ultimately had good outcomes after sustained efforts to manage trauma, many did not. Narratives from disengaged adolescents themselves demonstrate complex challenges and unmet needs arising from trauma. Given the extent of trauma, stigma, isolation and lacking social support contributing to disengagement, with extremes including household stigma, abuse or intimate partner violence, they described overwhelming barriers to access available services.

Some disengaged adolescents described support from counselling, friendly clinicians and peer mentors. ALHIV who had stopped taking ART or attending clinic nevertheless continued to seek support from peer mentors, who provided a critical pathway for re‐engagement. Narratives illustrated the complexity of unmet needs, however, and some adolescents expressed persistent hopelessness under the weight of trauma and mental health burdens.

“At times even when you go for counselling, some of them just really want to know what is happening with you but they won’t help you.… I would just come [to town] and say ‘hi’ to [a peer]. If he asked me, ‘Have you come to the clinic?’ I would tell him, ‘No, I just came to say hi to you guys and I’m on my way back.’” – 20 to 24yo F

### 3.6 Conceptual model: a cascade from trauma to disengagement

**Figure 2:**
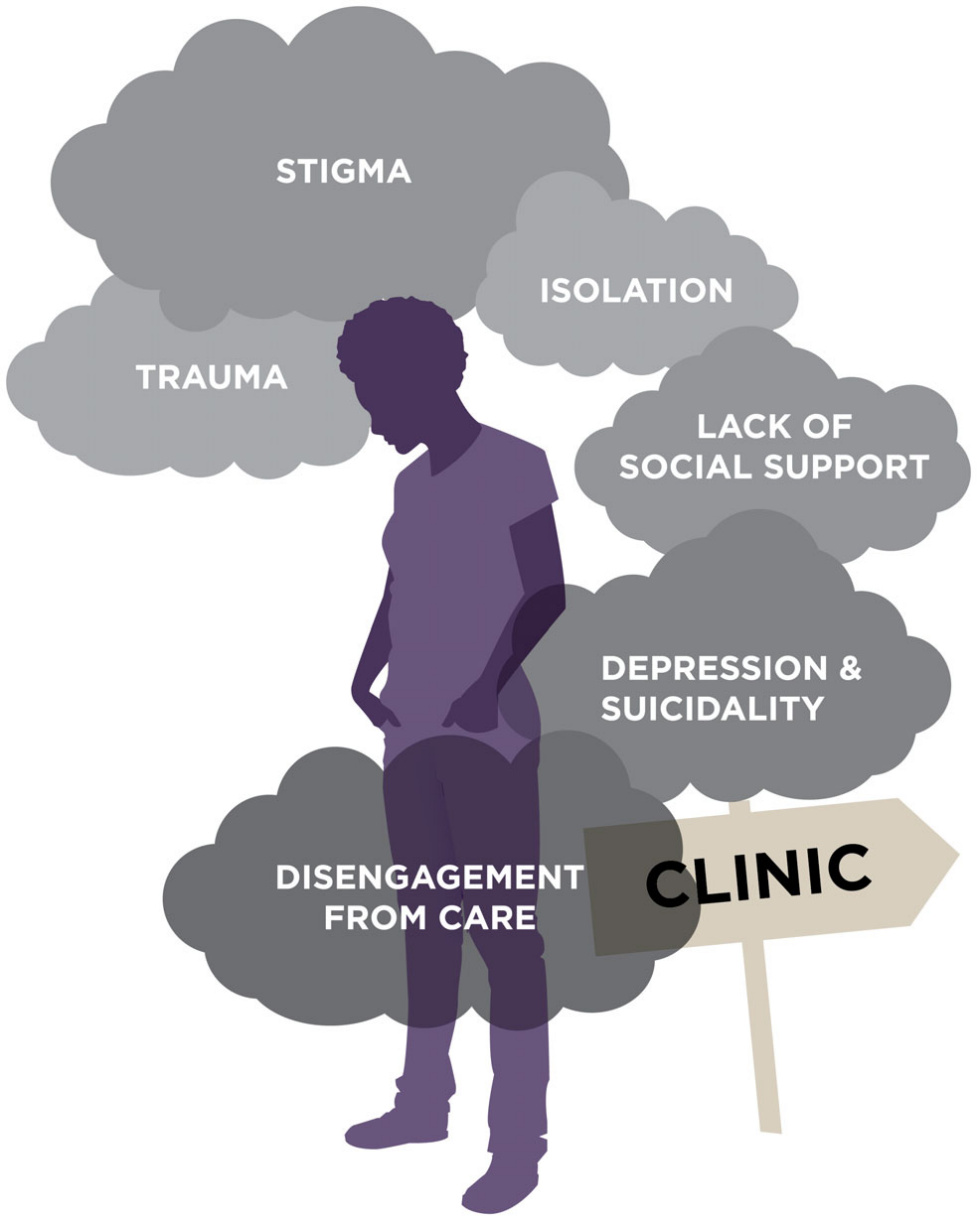
A conceptual model representing the cascade of factors from experiences of trauma to disengagement from care among adolescents living with HIV.

A conceptual model emerged, representing a common cascade from adolescent experience of trauma to disengagement from HIV care (Figure 2).

A *confluence of factors* compounded each other to make adolescent retention in HIV care untenable. Traumas (often multiple, repeated and/or violent) were compounded by experiences of stigma (which were themselves traumatic). Both elements could directly undermine care engagement through multiple pathways, such as when HIV care was associated with memories of parental loss or sexual assault, or when ongoing abuse or enacted stigma impeded care. Isolation resulted from the multiplicative and intersecting effects of trauma; enacted, anticipated and internalized stigma; and in some cases, abuse or neglect. Isolation was further compounded by lack of social support, particularly when adolescents were orphaned, or when they lacked stable familial or peer support. As a result, adolescents described experiences of profound hopelessness and depression. Hopelessness, in turn, was central to their decreased adherence and disengagement from care, in several cases reaching a point of suicidal ideation.

**Figure 3:**
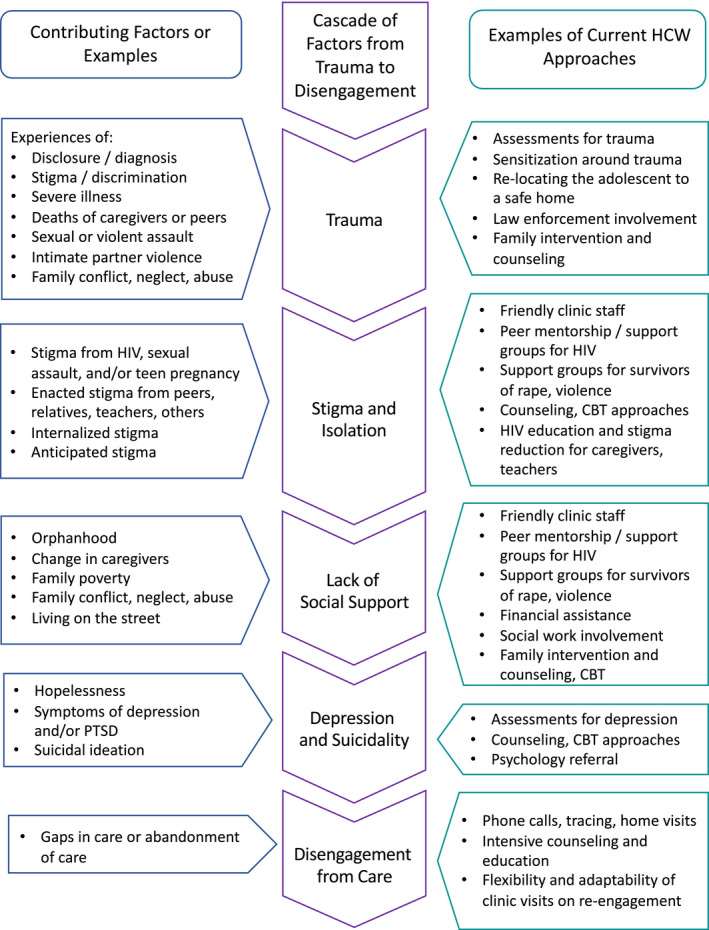
Example contributing factors and healthcare worker approaches within a cascade from trauma to disengagement among adolescents living with HIV.

HCW approaches to ALHIV coping with trauma aligned with the factors outlined by this model, suggesting that these areas may serve as targets for intervention and provision of trauma‐informed care (Figure 3).

## 4 DISCUSSION

Adolescents in this study experienced a high burden of traumatic experiences, particularly related to sexual and physical violence, enacted stigma, losses of parents or peers and severe illness. We found that the multiplicative and intersecting effects of trauma, stigma (enacted, anticipated and internalized) and lack of social support, resulted in hopelessness and disengagement. There emerged a cascade of factors which may represent key areas for intervention to support mental health and retention in HIV care. These include not only the provision of mental healthcare, but also preventing or addressing violence, trauma and stigma, and reinforcing social and familial support surrounding vulnerable adolescents. In this conceptualization, supporting retention in HIV care requires a trauma‐informed approach, both in the individualized care of ALHIV, and in the development of evidence‐based strategies, policies and resources to support adolescent health.

We observed a predominance of sexual and intimate partner violence impacting adolescents in this study. This included situations where sexual assault resulted in HIV infection, reflecting a critical target for prevention globally [31]. This finding is consistent with reports that sexual violence against adolescents in Kenya is high, impacting a range of health outcomes [32, 33, 34, 35, 36]. It is difficult to overstate the burden of sexual trauma among these adolescents who had disengaged from HIV care, and the unmet need for strategies to address it. We further noted that trauma was expressed predominantly among female ALHIV, and that this may reflect a disproportionate burden of trauma. Other contributions to this finding may include the greater number of female participants and/or cultural expectations that males may not express trauma readily.

This study is consistent with previous research that has found a predominance of sexual, physical violence and PTSD among women living with HIV; an association of trauma with poor outcomes; a cumulative effect of trauma on health outcomes; and important contributions of stigma, poverty, low social support and depression to poor HIV outcomes after trauma [23, 45]. Understanding pathways from trauma to poor health outcomes is an area of ongoing investigation [42]. This study adds to this work by elaborating adolescent narratives of trauma; triangulating these with caregiver and HCW narratives; elucidating pathways from trauma to disengagement and conceptualizing targets for intervention across multiple levels within a conceptual model.

Strategies are needed to provide trauma‐informed care to ALHIV [15, 46, 47, 48, 49, 50]. Key targets for intervention may centre on preventing, addressing, or mitigating each of the contributing impacts of trauma, stigma, lacking social support and mental health disorders. The cascade towards disengagement is not an inevitable progression; even after significant traumas, HCW described how their work in these areas supported adolescents coping with trauma to continue in care and achieve healthy outcomes. There is a need to study and expand interventions or packages that may work across multiple targets of this cascade to optimally support adolescents, including those who may not have disclosed underlying trauma.

Peer mentorship, for example is highlighted by this study as a critical intervention that addresses multiple levels of the cascade from trauma to disengagement. Peer mentorship mitigates against the stigma and isolation that adolescents’ experience, bolsters social connectedness and links adolescents to more intensive psychological counselling and support. As such, peer mentorship forms a crucial component of trauma‐informed care for adolescents, who may particularly rely on the lifeline that peers provide [6, 7, 51, 52]. Our findings suggest a potential role for peer mental health counselling, such as the “friendship bench” model, or group‐based mental health interventions [53, 54, 55, 56]. They also further bolster evidence for peer‐based care models for adolescent HIV care [6, 10, 57, 58, 59].

Adolescents in this study described significant mental health needs. Research has found massive underdiagnosis and undertreatment of mental health disorders in Kenya, as in other settings [60, 61]. There is an urgency to build up mental health services and capacity, including by developing a cadre of mental health professionals or supporters and investing in strategies to disseminate mental health interventions. While AMPATH provides mental healthcare, in this study we focused on disengaged adolescents, whose experiences illustrate gaps for highly vulnerable adolescents. These may relate to limitations in the provision of mental health services in contrast to the number of adolescents with underlying trauma or mental health disorders. Validated and culturally appropriate tools are needed for screening and management of trauma and depression among adolescents in HIV care settings [62]. Few studies have examined trauma interventions among individuals living with HIV, particularly in sub‐Saharan Africa. Cognitive behavioural therapy approaches and support groups may be promising, and have recently been studied in South Africa and Rwanda among women living with HIV with experiences of trauma and sexual violence [43, 63, 64, 65, 66].

Stigma and lack of social support are critical mediating factors to disengagement after trauma [67, 68, 69, 70]. Indeed, these challenges may underlie limited access to both mental health services and HIV care, despite the availability of quality services. Strategies are needed to bolster peer, familial or social support for ALHIV to enable them to access services for both HIV and mental healthcare [2, 71, 72]. These may include family therapy, as well as school‐ or community‐based interventions.

Because this analysis focused on trauma in narratives of disengagement, this work does not exhaust additional reasons for disengagement, nor does it reflect other dimensions of adolescents’ lives beyond HIV care, in which they may have been managing better [73, 74]. Our research objective to understand pathways from trauma to disengagement was facilitated by rigorous qualitative methods. The validity of our findings is supported by triangulation among disengaged adolescents, caregivers and HCW at a range of sites, whose narratives both supported and elaborated on each other. Furthermore, our analysis benefitted from close interrogation of the data and analysis by multiple investigators with experience with ALHIV in this setting. In addition, our findings are broadly consistent with those of other studies of ALHIV in sub‐Saharan Africa [6, 7, 9, 10, 11, 68]. Finally, a major strength of this study is the inclusion of ALHIV who have disengaged from care in in‐depth interviews, as their experiences and narratives are critical to understanding the challenges of retention in HIV care.

## 5 CONCLUSIONS

Trauma is a major factor underlying adolescent disengagement from HIV care. Among disengaged adolescents, high burdens of trauma were compounded by stigma, isolation and lack of social support. These factors contributed to worsened mental health, poor adherence and disengagement. This cascade represents key targets to prevent or respond to trauma; to support social, emotional and mental health; and to bolster care engagement and adherence for adolescents who have experienced a range of trauma. There is a crucial unmet need for mental health and social support among adolescents facing trauma and mental health challenges which greatly impact their ongoing care for HIV.

## COMPETING INTERESTS

The authors declare no conflicts of interest.

## AUTHORS’ CONTRIBUTIONS

LAE, EA, JDF, WMN, KWK, BE and RCV designed this study. MO and SB recruited and enrolled participants and performed interviews. LAE, EA and JA supervised the study. LAE performed all stages of the analysis and drafting of the manuscript. LAE, EA, MO, JJT, SB and CM coded and analysed transcripts. All authors participated in the analysis and in the revision of manuscript drafts. All authors have reviewed and approved the final manuscript.

## References

[1] UNAIDS . UNAIDS estimates 2020. Geneva: UNAIDS; 2020 [cited 2020 Aug 18]. Available from: https://aidsinfo.unaids.org/

[2] Casale M , Carlqvist A , Cluver L . Recent interventions to improve retention in HIV care and adherence to antiretroviral treatment among adolescents and youth: a systematic review. AIDS Patient Care STDS. 2019;33(6):237–52. 31166783 10.1089/apc.2018.0320 PMC6588099

[3] Desmonde S , Tanser F , Vreeman R , Takassi E , Edmonds A , Lumbiganon P , et al. Access to antiretroviral therapy in HIV‐infected children aged 0 to 19 years in the International Epidemiology Databases to Evaluate AIDS (IeDEA) Global Cohort Consortium, 2004–2015: a prospective cohort study. PLoS Med. 2018;15:e1002565. 29727458 10.1371/journal.pmed.1002565 PMC5935422

[4] Enane LA , Davies MA , Leroy V , Edmonds A , Apondi E , Adedimeji A , et al. Traversing the cascade: urgent research priorities for implementing the 'treat all' strategy for children and adolescents living with HIV in sub‐Saharan Africa. J Virus Erad. 2018;4 Suppl 2:40–6. 30515313 10.1016/S2055-6640(20)30344-7 PMC6248846

[5] Mokdad AH , Forouzanfar MH , Daoud F , Mokdad AA , El Bcheraoui C , Moradi‐Lakeh M , et al. Global burden of diseases, injuries, and risk factors for young people's health during 1990–2013: a systematic analysis for the Global Burden of Disease Study 2013. Lancet. 2016;387(10036):2383–401. 27174305 10.1016/S0140-6736(16)00648-6

[6] Enane LA , Apondi E , Toromo J , Bosma C , Ngeresa A , Nyandiko W , et al. "A problem shared is half solved" ‐ a qualitative assessment of barriers and facilitators to adolescent retention in HIV care in western Kenya. AIDS Care. 2020;32(1):104–12. 31554414 10.1080/09540121.2019.1668530 PMC6883166

[7] Enane LA , Mokete K , Joel D , Daimari R , Tshume O , Anabwani G , et al. "We did not know what was wrong"‐Barriers along the care cascade among hospitalized adolescents with HIV in Gaborone, Botswana. PLoS One. 2018;13:e0195372. 29630654 10.1371/journal.pone.0195372 PMC5890999

[8] Enane LA , Vreeman RC , Foster C . Retention and adherence: global challenges for the long‐term care of adolescents and young adults living with HIV. Curr Opin HIV AIDS. 2018;13(3):212–9. 29570471 10.1097/COH.0000000000000459

[9] Wolf HT , Halpern‐Felsher BL , Bukusi EA , Agot KE , Cohen CR , Auerswald CL . "It is all about the fear of being discriminated [against]..the person suffering from HIV will not be accepted": a qualitative study exploring the reasons for loss to follow‐up among HIV‐positive youth in Kisumu, Kenya. BMC Public Health. 2014;14:1154. 25377362 10.1186/1471-2458-14-1154 PMC4232620

[10] Zanoni BC , Sibaya T , Cairns C , Haberer JE . Barriers to retention in care are overcome by adolescent‐friendly services for adolescents living with HIV in south Africa: a qualitative analysis. AIDS Behav. 2019;23(4):957–65. 30535836 10.1007/s10461-018-2352-6 PMC6459720

[11] Williams S , Renju J , Ghilardi L , Wringe A . Scaling a waterfall: a meta‐ethnography of adolescent progression through the stages of HIV care in sub‐Saharan Africa. J Int AIDS Soc. 2017;20:21922. 28953326 10.7448/IAS.20.1.21922 PMC5640312

[12] Verhey R , Chibanda D , Vera A , Manda E , Brakarsh J , Seedat S . Perceptions of HIV‐related trauma in people living with HIV in Zimbabwe's Friendship Bench Program: a qualitative analysis of counselors' and clients' experiences. Transcult Psychiatry. 2020;57(1):161–72. 31180824 10.1177/1363461519850337

[13] Mgbako O , Benoit E , Iyengar NS , Kuhner C , Brinker D , Duncan DT . "Like a ticking time bomb": the persistence of trauma in the HIV diagnosis experience among black men who have sex with men in New York City. BMC Public Health. 2020;20(1):1247. 32807117 10.1186/s12889-020-09342-9 PMC7433074

[14] Watt MH , Dennis AC , Choi KW , Ciya N , Joska JA , Robertson C , et al. Impact of sexual trauma on hiv care engagement: perspectives of female patients with trauma histories in Cape Town, South Africa. AIDS Behav. 2017;21(11):3209–18. 27866288 10.1007/s10461-016-1617-1 PMC5438301

[15] Sales JM , Swartzendruber A , Phillips AL . Trauma‐informed HIV prevention and treatment. Curr HIV/AIDS Rep. 2016;13(6):374–82. 27704251 10.1007/s11904-016-0337-5 PMC5107145

[16] LeGrand S , Reif S , Sullivan K , Murray K , Barlow ML , Whetten K . A review of recent literature on trauma among individuals living with HIV. Curr HIV/AIDS Rep. 2015;12(4):397–405. 26419376 10.1007/s11904-015-0288-2 PMC4837695

[17] Villar‐Loubet OM , Illa L , Echenique M , Cook R , Messick B , Duthely LM , et al. Prenatal and mental health care among trauma‐exposed, HIV‐infected, pregnant women in the United States. J Assoc Nurses AIDS Care. 2014;25 1 Suppl:S50–61. 24274993 10.1016/j.jana.2013.06.006

[18] Soto T , Komaie G , Neilands TB , Johnson MO . Exposure to crime and trauma among HIV‐infected men who have sex with men: associations with HIV stigma and treatment engagement. J Assoc Nurses AIDS Care. 2013;24(4):299–307. 23790273 10.1016/j.jana.2012.11.008

[19] Kim MH , Mazenga AC , Yu X , Ahmed S , Paul ME , Kazembe PN , et al. High self‐reported non‐adherence to antiretroviral therapy amongst adolescents living with HIV in Malawi: barriers and associated factors. J Int AIDS Soc. 2017;20:21437. 28406275 10.7448/IAS.20.1.21437 PMC5515061

[20] Garner AS , Shonkoff JP , Siegel BS , Dobbins MI , Earls MF , Garner AS , McGuinn L , et al. Early childhood adversity, toxic stress, and the role of the pediatrician: translating developmental science into lifelong health. Pediatrics. 2012;129(1):e224–31. 22201148 10.1542/peds.2011-2662

[21] Oral R , Ramirez M , Coohey C , Nakada S , Walz A , Kuntz A , et al. Adverse childhood experiences and trauma informed care: the future of health care. Pediatr Res. 2016;79(1–2):227–33. 26460523 10.1038/pr.2015.197

[22] The National Child Traumatic Stress Network . About Child Trauma [cite 31 Jan 2021]. Available from: https://www.nctsn.org/what‐is‐child‐trauma/about‐child‐trauma

[23] Mugavero MJ , Raper JL , Reif S , Whetten K , Leserman J , Thielman NM , et al. Overload: impact of incident stressful events on antiretroviral medication adherence and virologic failure in a longitudinal, multisite human immunodeficiency virus cohort study. Psychosom Med. 2009;71(9):920–6. 19875634 10.1097/PSY.0b013e3181bfe8d2 PMC3691857

[24] The National Child Traumatic Stress Network . Trauma types. [cited 2021 Jan 31]. [Available from: https://www.nctsn.org/what‐is‐child‐trauma/trauma‐types

[25] Einterz RM , Kelley CR , Mamlin JJ , Van Reken DE . Partnerships in international health. The Indiana University‐Moi University experience. Infect Dis Clin North Am. 1995;9(2):453–5. 7673682

[26] Einterz RM , Kimaiyo S , Mengech HN , Khwa‐Otsyula BO , Esamai F , Quigley F , et al. Responding to the HIV pandemic: the power of an academic medical partnership. Acad Med. 2007;82(8):812–8. 17762264 10.1097/ACM.0b013e3180cc29f1

[27] Mercer T , Gardner A , Andama B , Chesoli C , Christoffersen‐Deb A , Dick J , et al. Leveraging the power of partnerships: spreading the vision for a population health care delivery model in western Kenya. Global Health. 2018;14(1):44. 10.1186/s12992-018-0366-5 29739421 PMC5941561

[28] Pastakia SD , Tran DN , Manji I , Schellhase E , Karwa R , Miller ML , et al. Framework and case study for establishing impactful global health programs through academia ‐ biopharmaceutical industry partnerships. Res Social Adm Pharm. 2020;16(11):1519–1525. 10.1016/j.sapharm.2020.07.018 32792324

[29] Braitstein P , Katshcke A , Shen C , Sang E , Nyandiko W , Ochieng VO , et al. Retention of HIV‐infected and HIV‐exposed children in a comprehensive HIV clinical care programme in Western Kenya. Trop Med Int Health. 2010;15(7):833–41. 20487430 10.1111/j.1365-3156.2010.02539.x PMC2929358

[30] Braitstein P , Songok J , Vreeman RC , Wools‐Kaloustian KK , Koskei P , Walusuna L , et al. "Wamepotea" (they have become lost): outcomes of HIV‐positive and HIV‐exposed children lost to follow‐up from a large HIV treatment program in western Kenya. J Acquir Immune Defic Syndr. 2011;57(3):e40–6. 21407085 10.1097/QAI.0b013e3182167f0d PMC3145828

[31] UNAIDS . UNAIDS Data 2020. Geneva; 2020.

[32] Ajema C , Mbugua C , Memiah P , Wood C , Cook C , Kotut R , et al. Addressing the dual health epidemics of HIV and sexual abuse among children and adolescents in Kenya: uptake of HIV counseling and post‐exposure prophylaxis. Adolesc Health Med Ther. 2018;9:1–9. 29296104 10.2147/AHMT.S149416 PMC5741064

[33] Atwoli L , Ayuku D , Hogan J , Koech J , Vreeman RC , Ayaya S , et al. Impact of domestic care environment on trauma and posttraumatic stress disorder among orphans in western Kenya. PLoS One. 2014;9:e89937. 24625395 10.1371/journal.pone.0089937 PMC3953071

[34] Baiocchi M , Friedberg R , Rosenman E , Amuyunzu‐Nyamongo M , Oguda G , Otieno D , et al. Prevalence and risk factors for sexual assault among class 6 female students in unplanned settlements of Nairobi, Kenya: Baseline analysis from the IMPower & Sources of Strength cluster randomized controlled trial. PLoS One. 2019;14:e0213359. 31170151 10.1371/journal.pone.0213359 PMC6553848

[35] Embleton L , Wachira J , Kamanda A , Naanyu V , Winston S , Ayuku D , et al. "Once you join the streets you will have to do it": sexual practices of street children and youth in Uasin Gishu County, Kenya. Reprod Health. 2015;12:106. 26573581 10.1186/s12978-015-0090-z PMC4647324

[36] Mathur S , Okal J , Musheke M , Pilgrim N , Kishor Patel S , Bhattacharya R , et al. High rates of sexual violence by both intimate and non‐intimate partners experienced by adolescent girls and young women in Kenya and Zambia: Findings around violence and other negative health outcomes. PLoS One. 2018;13:e0203929. 30212561 10.1371/journal.pone.0203929 PMC6136792

[37] Brief DJ , Bollinger AR , Vielhauer MJ , Berger‐Greenstein JA , Morgan EE , Brady SM , et al. Understanding the interface of HIV, trauma, post‐traumatic stress disorder, and substance use and its implications for health outcomes. AIDS Care. 2004;16 Suppl 1:S97–120. 15736824 10.1080/09540120412301315259

[38] Leserman J , Barroso J , Pence BW , Salahuddin N , Harmon JL . Trauma, stressful life events and depression predict HIV‐related fatigue. AIDS Care. 2008;20(10):1258–65. 18608079 10.1080/09540120801919410 PMC2603249

[39] Leserman J , Pence BW , Whetten K , Mugavero MJ , Thielman NM , Swartz MS , et al. Relation of lifetime trauma and depressive symptoms to mortality in HIV. Am J Psychiatry. 2007;164(11):1707–13. 17974936 10.1176/appi.ajp.2007.06111775

[40] Machtinger EL , Wilson TC , Haberer JE , Weiss DS . Psychological trauma and PTSD in HIV‐positive women: a meta‐analysis. AIDS Behav. 2012;16(8):2091–100. 22249954 10.1007/s10461-011-0127-4

[41] Mugavero M , Ostermann J , Whetten K , Leserman J , Swartz M , Stangl D , et al. Barriers to antiretroviral adherence: the importance of depression, abuse, and other traumatic events. AIDS Patient Care STDS. 2006;20(6):418–28. 16789855 10.1089/apc.2006.20.418

[42] Pence BW , Mugavero MJ , Carter TJ , Leserman J , Thielman NM , Raper JL , et al. Childhood trauma and health outcomes in HIV‐infected patients: an exploration of causal pathways. J Acquir Immune Defic Syndr. 2012;59(4):409–16. 22107822 10.1097/QAI.0b013e31824150bb PMC3299853

[43] Seedat S . Interventions to improve psychological functioning and health outcomes of HIV‐infected individuals with a history of trauma or PTSD. Curr HIV/AIDS Rep. 2012;9(4):344–50. 23007792 10.1007/s11904-012-0139-3

[44] Sledjeski EM , Speisman B , Dierker LC . Does number of lifetime traumas explain the relationship between PTSD and chronic medical conditions? Answers from the National Comorbidity Survey‐Replication (NCS‐R). J Behav Med. 2008;31(4):341–9. 18553129 10.1007/s10865-008-9158-3 PMC2659854

[45] Whetten K , Shirey K , Pence BW , Yao J , Thielman N , Whetten R , et al. Trauma history and depression predict incomplete adherence to antiretroviral therapies in a low income country. PLoS One. 2013;8:e74771. 24124455 10.1371/journal.pone.0074771 PMC3790775

[46] Brezing C , Ferrara M , Freudenreich O . The syndemic illness of HIV and trauma: implications for a trauma‐informed model of care. Psychosomatics. 2015;56(2):107–18. 25597836 10.1016/j.psym.2014.10.006

[47] Cuca YP , Shumway M , Machtinger EL , Davis K , Khanna N , Cocohoba J , et al. The association of trauma with the physical, behavioral, and social health of women living with HIV: pathways to guide trauma‐informed health care interventions. Womens Health Issues. 2019;29(5):376–84. 31303419 10.1016/j.whi.2019.06.001 PMC6755036

[48] Desilets L , Fernet M , Otis J , Cousineau MM , Massie L , De Pokomandy A , et al. Trauma‐informed practices to address intersections between HIV and intimate partner violence among women: perspective of community service providers. J Assoc Nurses AIDS Care. 2020;31(2):176–89. 32058333 10.1097/JNC.0000000000000163

[49] Kalokhe AS , Riddick C , Piper K , Schiff J , Getachew B , Del Rio C , et al. Integrating program‐tailored universal trauma screening into HIV care: an evidence‐based participatory approach. AIDS Care. 2020;32(2):209–16. 31357876 10.1080/09540121.2019.1640841 PMC10243449

[50] Tavakkoli M , Cohen MA , Alfonso CA , Batista SM , Tiamson‐Kassab ML , Meyer P . Caring for persons with early childhood trauma, PTSD, and HIV: a curriculum for clinicians. Acad Psychiatry. 2014;38(6):696–700. 25005006 10.1007/s40596-014-0186-8

[51] Bershetyn A , Odeny TA , Lyamuya R , Nakiwogga‐Muwanga A , Diero L , Bwana M , et al. The causal effect of tracing by peer health workers on return to clinic among patients who were lost to follow‐up from antiretroviral therapy in eastern Africa: a "natural experiment" arising from surveillance of lost patients. Clin Infect Dis. 2017;64(11):1547–54. 28329184 10.1093/cid/cix191 PMC5848300

[52] Shah P , Kibel M , Ayuku D , Lobun R , Ayieko J , Keter A , et al. a pilot study of "peer navigators" to promote uptake of HIV testing, care and treatment among street‐connected children and youth in Eldoret, Kenya. AIDS Behav. 2019;23(4):908–19. 30269232 10.1007/s10461-018-2276-1 PMC6458975

[53] Chibanda D . Reducing the treatment gap for mental, neurological and substance use disorders in Africa: lessons from the Friendship Bench in Zimbabwe. Epidemiol Psychiatr Sci. 2017;26(4):342–7. 28399952 10.1017/S2045796016001128 PMC6998766

[54] Chibanda D , Bowers T , Verhey R , Rusakaniko S , Abas M , Weiss HA , et al. The Friendship Bench programme: a cluster randomised controlled trial of a brief psychological intervention for common mental disorders delivered by lay health workers in Zimbabwe. Int J Ment Health Syst. 2015;9:21. 27408619 10.1186/s13033-015-0013-y PMC4940904

[55] Chibanda D , Verhey R , Munetsi E , Cowan FM , Lund C . Using a theory driven approach to develop and evaluate a complex mental health intervention: the friendship bench project in Zimbabwe. Int J Ment Health Syst. 2016;10:16. 26933448 10.1186/s13033-016-0050-1 PMC4772526

[56] Dow DE , Mmbaga BT , Gallis JA , Turner EL , Gandhi M , Cunningham CK , et al. A group‐based mental health intervention for young people living with HIV in Tanzania: results of a pilot individually randomized group treatment trial. BMC Public Health. 2020;20(1):1358. 32887558 10.1186/s12889-020-09380-3 PMC7487650

[57] Mavhu W , Willis N , Mufuka J , Bernays S , Tshuma M , Mangenah C , et al. Effect of a differentiated service delivery model on virological failure in adolescents with HIV in Zimbabwe (Zvandiri): a cluster‐randomised controlled trial. Lancet Glob Health. 2020;8(2):e264–75. 31924539 10.1016/S2214-109X(19)30526-1

[58] Stangl A , Bond V , Mackworth‐Young C , Sievwright K , Singh D , Clay S , et al. Transitioning to a Healthy Adulthood ‐ Adolescent Girls, HIV and AIDS. Lessons learned from adolescent girls living with HIV in urban, Zambia. 2015.

[59] Zanoni BC , Sibaya T , Cairns C , Lammert S , Haberer JE . Higher retention and viral suppression with adolescent‐focused HIV clinic in South Africa. PLoS One. 2017;12:e0190260. 29287088 10.1371/journal.pone.0190260 PMC5747481

[60] Kwobah E , Epstein S , Mwangi A , Litzelman D , Atwoli L . PREVALENCE of psychiatric morbidity in a community sample in Western Kenya. BMC Psychiatry. 2017;17(1):30. 28100210 10.1186/s12888-017-1202-9 PMC5242046

[61] Meyer A , Ndetei D . Providing Sustainable Mental Health Care in Kenya: A Demonstration Project. In: Forum on Neuroscience and Nervous System Disorders; Board on Health Sciences Policy; Board on Global Health; Institute of Medicine; National Academies of Sciences, Engineering, and Medicine. Providing Sustainable Mental and Neurological Health Care in Ghana and Kenya: Workshop Summary. Washington, DC; 2016 25 February 2016. 26447267

[62] Vreeman RC , McCoy BM , Lee S . Mental health challenges among adolescents living with HIV. J Int AIDS Soc. 2017;20(Suppl 3):21497. 28530045 10.7448/IAS.20.4.21497 PMC5577712

[63] Knettel BA , Mulawa MI , Knippler ET , Ciya N , Robertson C , Joska JA , et al. Women's perspectives on ImpACT: a coping intervention to address sexual trauma and improve HIV care engagement in Cape Town, South Africa. AIDS Care. 2019;31(11):1389–96. 30821168 10.1080/09540121.2019.1587368 PMC6717688

[64] Knettel BA , Robertson C , Ciya N , Coleman JN , Elliott SA , Joska JA , et al. "I cannot change what happened to me, but I can learn to change how I feel": A case study from ImpACT, an intervention for women with a history of sexual trauma who are living with HIV in Cape Town, South Africa. Psychotherapy (Chic). 2020;57(1):90–6. 31855042 10.1037/pst0000263 PMC7069791

[65] Sikkema KJ , Mulawa MI , Robertson C , Watt MH , Ciya N , Stein DJ , et al. Improving AIDS care after trauma (ImpACT): pilot outcomes of a coping intervention among HIV‐infected women with sexual trauma in South Africa. AIDS Behav. 2018;22(3):1039–52. 29270789 10.1007/s10461-017-2013-1 PMC5828984

[66] Walstrom P , Operario D , Zlotnick C , Mutimura E , Benekigeri C , Cohen MH . 'I think my future will be better than my past': examining support group influence on the mental health of HIV‐infected Rwandan women. Glob Public Health. 2013;8(1):90–105. 22812728 10.1080/17441692.2012.699539 PMC5576858

[67] Anderson JD , Li X , Qiao S , Zhou Y , Shen Z . The mediating effects of functions of social support on HIV‐related trauma and health‐related quality of life for PLHIV in China. AIDS Care. 2020;32(6):673–80. 31174430 10.1080/09540121.2019.1622633

[68] Boyes ME , Pantelic M , Casale M , Toska E , Newnham E , Cluver LD . Prospective associations between bullying victimisation, internalised stigma, and mental health in South African adolescents living with HIV. J Affect Disord. 2020;276:418–23. 32871672 10.1016/j.jad.2020.07.101

[69] Rodriguez VJ , Butts SA , Mandell LN , Weiss SM , Kumar M , Jones DL . The role of social support in the association between childhood trauma and depression among HIV‐infected and HIV‐uninfected individuals. Int J STD AIDS. 2019;30(1):29–36. 30170529 10.1177/0956462418793736

[70] Rzeszutek M , Oniszczenko W , Zebrowska M , Firlag‐Burkacka E . HIV infection duration, social support and the level of trauma symptoms in a sample of HIV‐positive Polish individuals. AIDS Care. 2015;27(3):363–9. 25296635 10.1080/09540121.2014.963018

[71] Casale M , Boyes M , Pantelic M , Toska E , Cluver L . Suicidal thoughts and behaviour among South African adolescents living with HIV: Can social support buffer the impact of stigma? J Affect Disord. 2019;245:82–90. 30368074 10.1016/j.jad.2018.10.102

[72] Cluver LD , Toska E , Orkin FM , Meinck F , Hodes R , Yakubovich AR , et al. Achieving equity in HIV‐treatment outcomes: can social protection improve adolescent ART‐adherence in South Africa? AIDS Care. 2016;28 Suppl 2:73–82. 27392002 10.1080/09540121.2016.1179008 PMC4991216

[73] Bernays S , Paparini S , Seeley J , Rhodes T . "Not Taking it Will Just be Like a Sin": young people living with HIV and the stigmatization of less‐than‐perfect adherence to antiretroviral therapy. Med Anthropol. 2017;36(5):485–99. 28379042 10.1080/01459740.2017.1306856

[74] Horter S , Seeley J , Bernays S , Kerschberger B , Lukhele N , Wringe A . Dissonance of choice: biomedical and lived perspectives on HIV treatment‐taking. Med Anthropol. 2020;39(8):675–88. 32078396 10.1080/01459740.2020.1720981

